# Novel MtCEP1 peptides produced *in vivo* differentially regulate root development in *Medicago truncatula*

**DOI:** 10.1093/jxb/erv008

**Published:** 2015-02-22

**Authors:** Nadiatul A. Mohd-Radzman, Steve Binos, Thy T. Truong, Nijat Imin, Michael Mariani, Michael A. Djordjevic

**Affiliations:** ^1^Division of Plant Sciences, Research School of Biology, College of Medicine, Biology and Environment, The Australian National University, Canberra ACT 0200, Australia; ^2^Thermo Fisher Scientific Pty Ltd, 5 Caribbean Drive, Scoresby, VIC 3179, Australia; ^3^Mass Spectrometry Facility, Research School of Biology, College of Medicine, Biology and Environment, The Australian National University, Canberra ACT 0200, Australia

**Keywords:** Legume root development, mass spectrometry, nodulation, peptide isolation, peptide signaling, post-translational modification, secreted peptides.

## Abstract

Small, post-translationally modified and secreted peptides regulate diverse plant developmental processes. Due to low natural abundance, it is difficult to isolate and identify these peptides. Using an improved peptide isolation protocol and Orbitrap mass spectrometry, nine 15-amino-acid CEP peptides were identified that corresponded to the two domains encoded by *Medicago truncatula CEP1* (*MtCEP1*). Novel arabinosylated and hydroxylated peptides were identified in root cultures overexpressing *MtCEP1*. The five most abundant CEP peptides were hydroxylated and these species were detected also in low amounts in vector control samples. Synthetic peptides with different hydroxylation patterns differentially affected root development. Notably, the domain 1 peptide hydroxylated at Pro4 and Pro11 (D1:HyP4,11) imparted the strongest inhibition of lateral root emergence when grown with 5mM KNO_3_ and stimulated the highest increase in nodule number when grown with 0mM KNO_3_. Inhibition of lateral root emergence by D1:HyP4,11 was not alleviated by removing peptide exposure. In contrast, the domain 2 peptide hydroxylated at Pro11 (D2:HyP11) increased stage III–IV lateral root primordium numbers by 6-fold (*P* < 0.001) which failed to emerge. Auxin addition at levels which stimulated lateral root formation in wild-type plants had little or no ameliorating effect on CEP peptide-mediated inhibition of lateral root formation or emergence. Both peptides increased and altered the root staining pattern of the auxin-responsive reporter *GH3:GUS* suggesting CEPs alter auxin sensitivity or distribution. The results showed that CEP primary sequence and post-translational modifications influence peptide activities and the improved isolation procedure effectively and reproducibly identifies and characterises CEPs.

## Highlight

Nine different hydroxylated and triarabinosylated species of *Medicago truncatula* MtCEP1 peptides were identified. Differential MtCEP1 hydroxylation patterns affected lateral organ initiation and formation, which could not be rescued by auxin.

## Introduction

Root development is mediated through complex pathways involving endogenous signalling molecules and external environmental stimuli (Van Norman *et al.*, 2011; Matsubayashi, 2012; Murphy *et al.*, 2012; Meng, 2012; Czyzewicz *et al.*, 2013). These endogenous signalling molecules include small secreted peptides which regulate lateral root development, nodule formation and root meristem maintenance (Matsubayashi, 2011; Delay *et al.*, 2013a; Mohd-Radzman *et al.*, 2013). Examples include CLE40 (from the CLAVATA3/EMBRYO SURROUNDING REGION family), which inhibits cell differentiation in the primary root meristem (Stahl *et al.*, 2009), the RGF (ROOT GROWTH FACTOR)/GLV (GOLVEN)/CLEL (CLE-like) family, which promotes meristem maintenance of the primary root and negatively regulates lateral root growth (Matsuzaki *et al.*, 2010; Meng *et al.*, 2012; Whitford *et al.*, 2012; Fernandez *et al.*, 2013) and MtCLE12 and 13 (and their legume orthologues), which negatively regulate root nodule development (Mortier *et al.*, 2010; Reid *et al.*, 2011; Saur *et al.*, 2011; Okamoto *et al.*, 2013). CEPs (*C*-TERMINALLY ENCODED PEPTIDEs) negatively regulate lateral and primary root development and positively regulate nodulation (Ohyama *et al.*, 2008; Delay *et al.*, 2013b; Imin *et al.*, 2013; Roberts *et al.*, 2013; Tabata *et al.*, 2014). These small, secreted peptides are cleaved from a precursor, post-translationally modified and secreted to the apoplast (Matsubayashi, 2012). Both the final size of processed peptide and its post-translational modification (PTM) greatly influence biological activity (Ohyama et al., 2008, 2009; Matsuzaki *et al.*, 2010; Whitford *et al.*, 2012).

Only a handful of putative secreted peptides have been conclusively shown to be secreted and identified as mature peptides with their PTMs (Ito *et al.*, 2006; Amano *et al.*, 2007; Shinohara and Matsubayashi, 2010; Ohki *et al.*, 2011; Whitford *et al.*, 2012; Okamoto *et al.*, 2013). Isolation and identification of secreted peptides is difficult due to their very low abundance *in vivo*. To address this, secreted peptide coding genes are commonly overexpressed in calli, cell cultures, hairy roots or whole plants to elevate mature peptide amounts (Amano *et al.*, 2007; Shinohara and Matsubayashi, 2010; Ohki *et al.*, 2011; Whitford *et al.*, 2012; Okamoto *et al.*, 2013) followed by the selective precipitation of the peptide-of-interest, purification using high-performance liquid chromatography (HPLC) and identification and characterization using mass spectrometry (MS). This procedure has identified hydroxylated and arabinosylated CLV3 and LjCLE-RS2 peptides (Kondo *et al.*, 2006; Ohyama *et al.*, 2009) and sulfated RGF/GOLVEN peptides (Matsuzaki *et al.*, 2010; Whitford *et al.*, 2012). Although many members of plant peptide families encode more than one peptide domain (Oelkers *et al.*, 2009; Delay *et al.*, 2013b; Imin *et al.*, 2013; Roberts *et al.*, 2013), to date, only a few peptides from multiple domain coding genes have been identified (Pearce *et al.*, 2001; Chen *et al.*, 2008; Tabata *et al.*, 2014).

PTMs of small secreted peptides are important for their biological activity (Matsubayashi, 2012). For CLE and CEP peptides, proline residues are hydroxylated to form 4-hydroxylproline (Ohyama *et al.*, 2008; Kondo *et al.*, 2011; Matsubayashi, 2012). Nuclear magnetic resonance (NMR) studies demonstrated that proline hydroxylation and triarabinosylation influences peptide flexibility and its ability to interact with its corresponding receptor (Kondo *et al.*, 2011; Bobay *et al.*, 2013; Shinohara and Matsubayashi, 2013). Peptides lacking PTMs are either inactive or less active than their modified counterparts (Ohyama *et al.*, 2009; Matsuzaki *et al.*, 2010; Whitford *et al.*, 2012; Imin *et al.*, 2013). Hence, determining the *in vivo* forms of secreted peptides in plants and validating their biological activities is important.

CEP peptides regulate root development in *Arabidopsis* and *Medicago* (Ohyama *et al.*, 2008; Delay *et al.*, 2013b; Imin *et al.*, 2013; Roberts *et al.*, 2013). In *Arabidopsis*, *AtCEP1* and *AtCEP3* reduce primary root growth and emerged lateral root number when these genes are overexpressed or when the corresponding peptides are applied to wild-type roots (Ohyama *et al.*, 2008; Delay *et al.*, 2013b) even though neither gene appears to be expressed in the root tip (Roberts *et al.*, 2013). In addition, an *Atcep3* mutant showed greater primary root growth and emerged lateral root number when grown under abiotic stress conditions (Delay *et al.*, 2013b). Tabata *et al.* (2014) very recently identified CEP receptors in *Arabidopsis* and proposed that perception of root derived CEP peptides by these shoot receptors mediates systemic nitrogen-demand signalling. In *Medicago*, *MtCEP1* expresses in the primary root tip, vascular tissue and lateral organ primordia particularly under nitrogen limitations (Imin *et al.*, 2013). *MtCEP1*, which encodes two peptide domains, modulates lateral organ formation in *Medicago* but does not affect root tip growth (Imin *et al.*, 2013). The *MtCEP1ox* roots (*35S:MtCEP1*) showed a reduction in emerged lateral root number, enhanced nodule number and formed periodic circumferential cell proliferation (CCP) sites (Imin *et al.*, 2013). At CCP sites, the root diameter is increased up to 40% due in part to roots containing an extra cortical cell layer (Imin *et al.* 2013). Putative MtCEP1 peptides synthesized with hydroxylation imparted the same phenotypes on wild-type roots as *MtCEP1ox* (Imin *et al.*, 2013). However, the endogenous mature peptides of MtCEP1 domain 1 and 2 have not been characterized.

In this study, we developed improved methodologies to sufficiently enrich and characterize MtCEP1 peptides *in vivo*. The transgenic root cultures overexpressing *MtCEP1* showed several of the phenotypes induced by overexpressing *MtCEP1* in transgenic plants. After validating the presence of secreted CEP peptides by bioassaying the secreted material, the MtCEP1 peptides present in the culture concentrates were examined and determined by MS. Differentially hydroxylated CEP peptides were the most abundant species produced. The effects on lateral organ formation of synthetic hydroxylated peptides corresponding to the abundant species found were assessed. A root clearing technique was used to quantify the stages of lateral root formation. Given the central role played by auxin in lateral organ formation, interactions between auxin and CEP peptides were examined. The ability of the synthetic auxin, 1-naphthaleneacetic acid (NAA), to rescue the CEP peptide-mediated inhibition of lateral root formation and emergence was determined. The auxin-responsive reporter *GH3:GUS* was also used to observe the effects of the CEP peptides on auxin distribution/sensitivity *in vivo*.

## Materials and methods

### Establishing *Medicago* root culture

Root transformation was carried out using *Agrobacterium rhizogenes* ArQUA1 cells containing the empty vector control pK7WG2D.1 (hereafter referred to as ‘vector control’) (Boisson-Dernier *et al.*, 2001) or pK7WG2D.1 containing *35S:MtCEP1* (Imin *et al.*, 2013). The transformed hairy roots were selected using kanamycin and screened for the GFP-visible marker. These composite plants were also scored for lateral root number and periodic CCP site formation which are major phenotypes of *MtCEP1* overexpression. Transgenic roots were then excised and grown on solid Fåhraeus medium containing 100mg/L cefotaxime and 1% sucrose in the dark at 25°C and sub-cultured every week until axenic. The transgenic roots were then transferred to liquid Fåhraeus medium and grown in the dark at 25°C with continuous shaking at 100rpm for 14 days prior to exudate collection. The phenotypes induced by *MtCEP1ox* transgenic roots were assessed.

### Modified extraction and isolation of secreted endogenous peptides

Culture exudates (150mL/flask) were filtered through 100 µm nylon mesh and concentrated 10 times by rotary evaporation prior to *ο*-chlorophenol/acetone precipitation as described by Ohyama *et al.* (2008). Centrifugation was conducted at 9000 *g* for two hours instead of at 10 000 *g* for 10min to improve peptide precipitation. The pellet was dissolved in 500 µL water and the solution was run through a PD MidiTrap G-10 size exclusion gravity column (exclusion limit >700 Mr, GE Healthcare Life Sciences). The column is first equilibrated with 16mL of 100mM ammonium acetate (pH 7) prior to sample addition. The peptide fraction was then washed off the column with 1.2mL of 100mM ammonium acetate. The peptide wash was lyophilized overnight and resuspended in 400 µL of 3% acetonitrile with 0.1% formic acid prior to analysis using Q Exactive Plus nano-LC-ESI-MS/MS (Thermo Fisher Scientific, Waltham, MA, USA). For the nano-LC-Chip-ESI-MS/MS (Agilent Technologies, Santa Clara, CA, USA) analysis, the samples were resuspended in 20 µL of 10% acetonitrile/water containing 0.1% formic acid.

### Assessing peptide biological activities

*Medicago truncatula* seeds were surface sterilized, stratified and germinated on Fåhraeus medium plates (Kusumawati *et al.*, 2008). To assess the biological activity of the hairy root exudates, the concentrated exudate solution was added to the Fåhraeus medium with 5mM KNO_3_ at 1% of the final concentration of the original exudates and the phenotypes induced were assessed. To assess the biological activity of the synthetic peptides, stock solutions corresponding to the five identified hydroxylated peptide species were added to the medium at 1 µM final concentration. For the peptide assay, six seedlings per plate were grown on Fåhraeus medium with 5mM KNO_3_ for 10 days. To assess the effect on root of the temporal exposure to CEP peptides, the 14-day-old harvested seedlings were first grown on CEP peptide-containing medium (1 μM) with 5mM KNO_3_ for three, five or nine days prior to transferring to 5mM KNO_3_ Fåhraeus medium with no added peptide. The position of the root tip of each seedling was marked right after the transfer to delineate peptide exposed from non-peptide exposed root. For the nodulation assays, the plants were grown on nitrogen-free Fåhraeus medium containing the respective synthetic peptides for four days prior to inoculation with *Sinorhizobium meliloti* strain WSM1022. The nodules were counted two weeks post-inoculation. For the NAA treatment, the seedlings were grown on 5mM KNO_3_ Fåhraeus medium with NAA added to the respective final concentrations. The *GH3:GUS* seedlings were grown on 5mM KNO_3_ Fåhraeus medium with or without synthetic peptide (1 µM final concentration). All the seedlings were grown at 20°C with a 16 hour photoperiod and a photon flux density of 100 µmol m^-2^ s^-1^.

### Beta-glucuronidase (GUS) staining and root sectioning

GUS activity was localized in transgenic plants carrying the *GH3:GUS* construct. The staining protocol was adapted from Vitha *et al.* (1995). The roots were first fixed with ice-cold fixative of 4% formaldehyde in phosphate buffer (pH 7) and subsequently washed three times within 60 minutes with ice-cold phosphate buffer. The X-gluc substrate solution (1mg 5-bromo-4-chloro-3-indolyl β-D-Glucuronide in 0.1mL methanol, 1mL phosphate buffer, 20 µL 0.1M potassium ferrocyanide, and 20 µL 0.1M potassium ferricyanide) was then vacuum infiltrated into the root tissue before overnight incubation at 37°C. The roots were then embedded in 3% DNA grade agarose and sectioned using a vibratome (1000 Plus; Vibratome Company). Staining was performed twice, each with six individual plants and observed using a Nikon SMZ1500 stereomicroscope (Nikon Inc., New York, USA).

### Peptide synthesis

Peptides were synthesized by GL BioChem Pty Ltd. (Shanghai, China) and validated (Djordjevic *et al.*, 2011).

### Quantification of *Medicago* root primordia by whole root clearing

Roots from 10-day-old plants were collected and incubated in 0.4% HCl and 20% methanol at 60°C for one hour and transferred to a solution containing 7% NaOH (w/v) and 60% ethanol for 30min. Roots were rehydrated with 40%, 20% and 10% ethanol, each for 10min, respectively at room temperature and vacuum infiltrated with 5% ethanol and 25% glycerol for 15min prior to mounting in the same solution for interference contrast microscopy (Leica DM 5500 B; Leica Microsystems).

### Identification and quantification with the Q Exactive Plus nano-LC-ESI-MS/MS

A Thermo Scientific Easy-nLC 1000 HPLC system was used in a two column configuration for separation of the concentrated peptide-enriched extracts. The extracts were initially loaded onto a Thermo Acclaim PepMap C18 trap reversed-phase column (75 µm x 2cm nanoviper, 3 µm particle size) at a maximum pressure setting of 800 bar. Separation was achieved at 300 nL/min using buffer A (0.1% formic acid in water) and buffer B (0.1% formic acid in acetonitrile) as mobile phases for gradient elution with a 75 µm x 25cm PepMap RSLC C18 (2 µm particle size) Easy-Spray Column at 35°C. Peptide elution employed a 3–10% acetonitrile gradient for 10min followed by 10–38% acetonitrile gradient for 47min. The total acquisition time, including a 95% acetonitrile wash and re-equilibration, was 70min. For each run, 7 µL of the pre-diluted samples from the *MtCEP1* overexpressed and vector control root exudates were injected. Two blank runs were included between each sample to minimize carryover to negligible levels.

The eluted peptides from the C18 column were introduced to the mass spectrometer via nano-ESI and analysed using the Q-Exactive Plus (Thermo Fisher Scientific, Waltham, MA, USA). The electrospray voltage was 1.8kV, and the ion transfer tube temperature was 275°C. Employing a top 10 ddMS2 acquisition method with preference for a specified target list of +1, +2 and +3 charged species (Supplementary Method 1), full MS scans were acquired in the Orbitrap mass analyzer over the range m/z 350–1800 with a mass resolution of 70 000 (at m/z 200). The target value was 1.00E+06 counts. The 10 most intense peaks with a charge state ≥1 were fragmented in the high energy C-trap dissociation collision cell with a normalized collision energy of 27% and tandem mass spectra were acquired in the Orbitrap mass analyzer with a mass resolution of either 17 500 or 35 000 at a m/z of 200. The AGC (Automatic Gain Control) target value in both instances was set to 5.0E+04 counts. The ion selection threshold was 1.00E+04 counts at 17.5K and 4.50E+03 counts at 35K resolution. The maximum allowed ion accumulation times was 30ms for full MS scans and 50 and 110ms for tandem mass spectra at 17.5 and 35K, respectively. For all the experiments, the dynamic exclusion time was set to 10 s.

Database searching of all.raw files was performed with Proteome Discoverer 1.4 (Thermo Fisher Scientific) initially using SEQUEST HT for searching against an annotated *M. truncatula* database. Database searching against the corresponding reversed database was also performed to evaluate the false discovery rate of peptide identification. The SEQUEST HT search parameters included a precursor ion mass tolerance +/-10 ppm and product ion mass tolerance of 0.08 m/z units. Cysteine carbamidomethylation was set as a fixed modification, while M, K and P oxidation, C-terminal amidation and deamidation (of NQ) as well as *N*-terminal Gln to pyro-Glu were set as variable modifications.

For relative quantification, serial dilutions of the synthetic peptide as a standard were injected equating to 20amol to 200 femtomol on column. The extracted ion chromatogram (+/- 5 ppm,) for each precursor m/z was used for calculating the relative amount of each species. A calibration curve was generated from the dilution series and the concentration of respective peptide species was extrapolated from the curve.

## Results

### Modified methodology to identify and quantify root-derived endogenous peptides

**Fig. 1.**
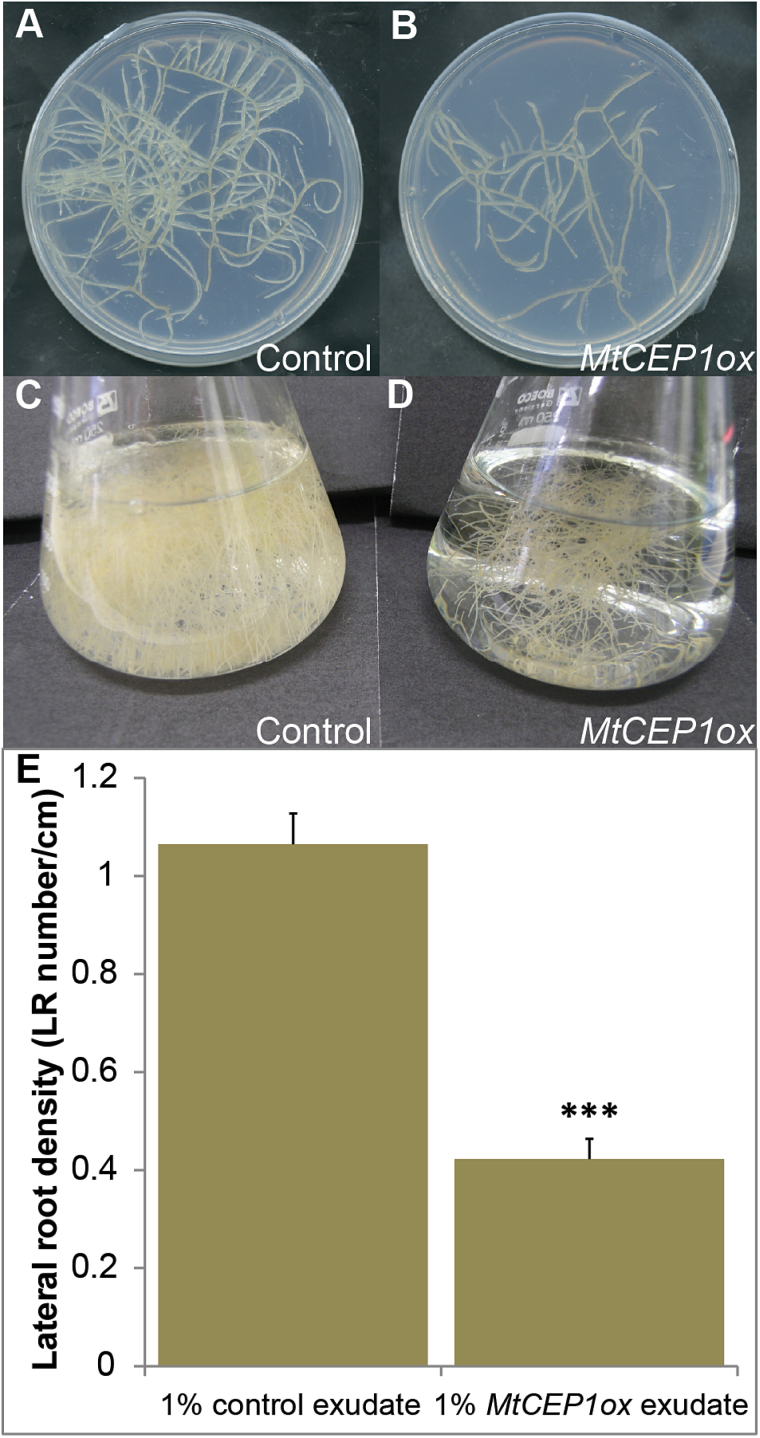
Strategy for enriching and bioassaying MtCEP1 peptides using root cultures. (A–D) Axenic root cultures containing either the vector (A, C) or *MtCEP1ox* (B, D). Root cultures were maintained on solidified Fåhraeus medium (A, B) prior to sub-culturing in liquid Fåhraeus medium (C, D) for peptide isolation. (A) and (B) show one week’s growth after adding an equally-sized root segment to the agar plates and (C) and (D) show two week’s growth in the liquid medium after equally sized root segments were initially sub-cultured. (E) The concentrated exudates from vector control and *MtCEP1ox* root cultures were incorporated into Fåhraeus medium to a final concentration of 1% of that present in the harvested flasks and bioassayed for inhibition of emerged lateral root number on wild type A17 seedlings (measured as LR number/cm) n=12.

To isolate the MtCEP1 mature peptides, axenic transformed root cultures were established using the same *MtCEP1ox* and vector control constructs reported in Imin *et al.* (2013) (Fig. 1A–D). Upon initial agar or liquid subculture, the starting root mass of the vector control and *MtCEP1ox* transgenic roots was the same. However, upon growth the *MtCEP1ox* root cultures formed significantly less root branches (~40% of the control; Student’s t-test, *P* < 0.001) resulting in a reduced root mass (Fig. 1 B, D). The *MtCEP1ox* cultures showed similar phenotypes to those previously observed in roots transformed with *MtCEP1ox* (i.e. a reduced number of lateral roots, CCP site formation and reduced root mass). To validate that biologically active secreted peptides were present in the culture medium, the root exudates from *MtCEP1ox* and vector control liquid cultures were collected, concentrated and added to the growth medium supporting the growth of wild-type *M. truncatula* seedlings to assay for their biological activities. The *MtCEP1ox* exudates reduced lateral root number indicating the presence of sufficient biologically active peptide in the liquid culture (Fig. 1E).

Current peptide isolation strategies (Ohyama *et al.*, 2008) utilize ο-chlorophenol precipitation, preparative HPLC and LC-electrospray ionization (ESI) ion trap MS for peptide identification and characterization. Since small peptide identification is challenging and the amino acids of MtCEP1 peptides lack a strong UV chromophore, the protocol was modified to enhance peptide detection by applying (i) a longer centrifugation time for *ο*-chlorophenol/acetone precipitation; (ii) a size exclusion gravity column (exclusion limit >700 Mr) for simultaneous isolation, buffer exchange, desalting and fast clean-up of the precipitated peptides; (iii) an additional lyophilization step to further concentrate the eluted peptides; and (iv) a sensitive high resolution, accurate mass nano-HPLC ESI with tandem MS using either the ChipCube ion source for the Q-TOF or the Q Exactive Plus Hybrid Quadrupole-Orbitrap mass spectrometer. A 10 000-fold concentration of the peptides in the sample was achieved using this experimental design. With the highly sensitive Quadrupole-Orbitrap MS, we successfully identified nine endogenous MtCEP1 peptides from the *MtCEP1ox* sample.

### The two CEP domains encoded by MtCEP1 are processed as 15-amino-acid peptides with post-translational modifications

**Fig. 2.**
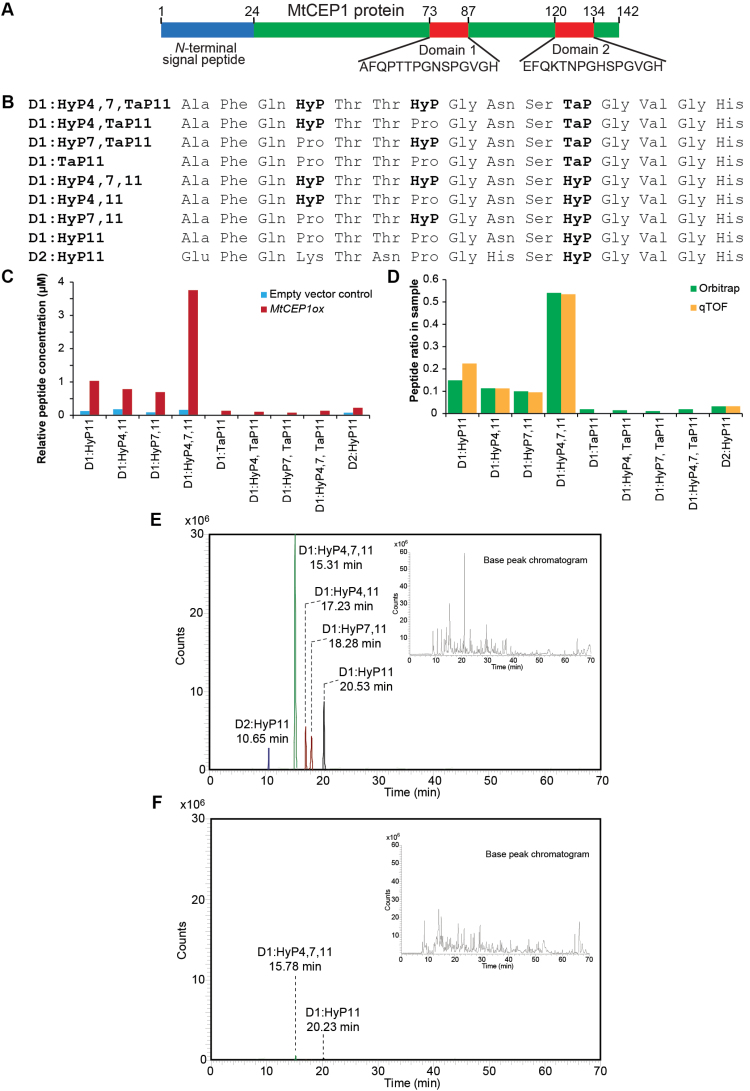
Identification of MtCEP1 peptide species in *MtCEP1ox* root and vector control exudates. (A) The pre-propeptide structure of MtCEP1 showing the two 15 amino acid peptide domains. (B) Eight species of MtCEP1 domain 1 (D1) peptide and one species of the domain 2 (D2) peptide were identified with their respective PTMs. HyP: hydroxylated proline; TaP: tri-arabinosylated proline. (C) The relative concentration of the nine MtCEP1 peptide species found in *MtCEP1ox* exudate and the five species found in the vector control exudate. Serial dilutions of the synthetic peptide were performed to establish a standard calibration curve (20amol to 200 femtomol) from which the concentration of each peptide was extrapolated. (D) The peptide species in *MtCEP1ox* samples were analysed using Quadrupole-Orbitrap and Q-TOF mass spectrometers. The ratio of the five peptide species identified correlated well between both nano-LC-ESI-MS systems. (E) The five most abundant peptides in *MtCEP1ox* sample eluted from the column based on their hydrophobicity as indicated by their retention time in the Quadrapole-Orbitrap. (F) The peptides in the vector control samples were detected in relative minute amounts as shown in the extracted ion chromatogram.

**Fig. 3.**
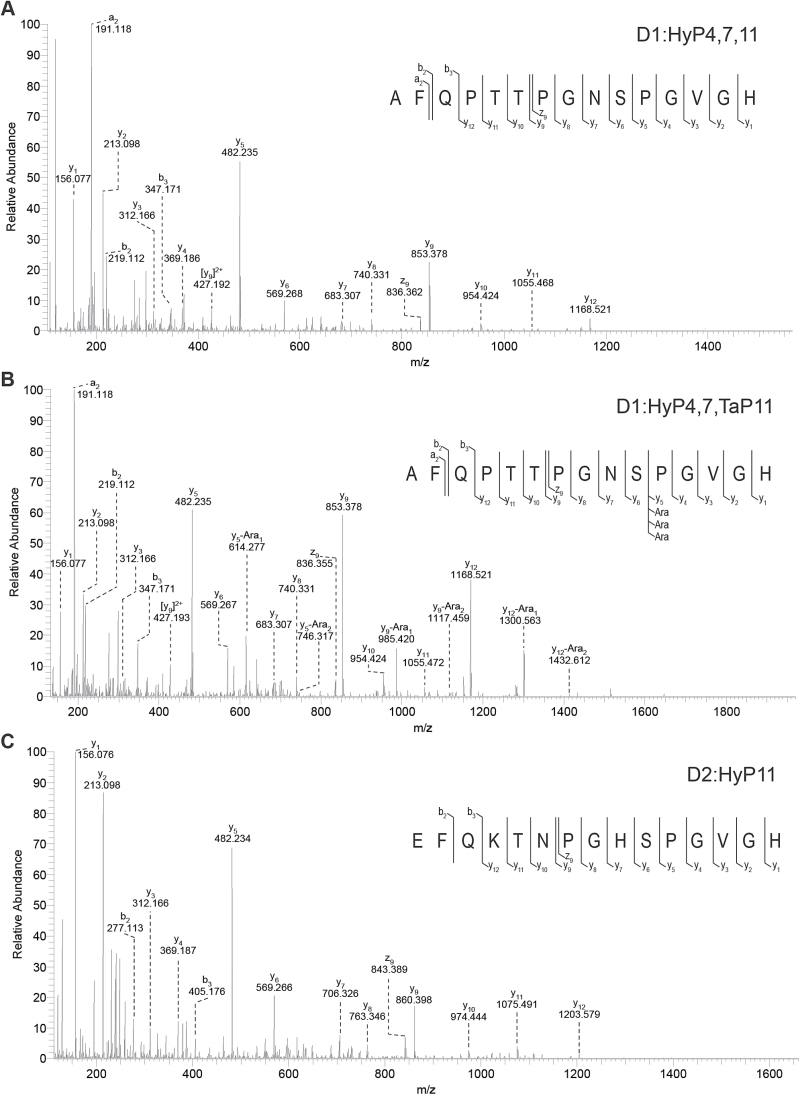
The MS/MS spectra of novel MtCEP1 peptide species analysed by Q Exactive Plus Hybrid Quadrupole-Orbitrap MS. (A) Identification of the trihydroxylated D1 peptide. Nano LC-MS/MS spectrum at 15.31min of the [M+2H]^2+^ for the trihydroxylated D1:HyP4,7,11 (m/z 757.8471) showed three signature ion fragments y5, y9 and y12 indicating that the 15 amino acid MtCEP1 D1 peptide is hydroxylated at all three prolines. (B) Identification of the dihydroxylated and tri-arabinosylated D1 peptide. The MS/MS spectrum at 16.11min of [M+2H]^2+^ for D1:HyP4,7,Tap11 (m/z 955.9105) showed extra peaks accompanying the three signature ion fragments with a shift of m/z 132 indicating that the peptide is arabinosylated at Pro11 and hydroxylated at Pro4 and Pro7. (C) Identification of the monohydroxylated D2 peptide. The MS/MS spectrum at 10.60min [M+2H]^2+^ for D2:HyP11 (m/z 804.3817) showed the 15 amino acid MtCEP1 D2 peptide is hydroxylated at Pro11. (This figure is available in colour at *JXB* online.)

Thus far, most of the successfully isolated and characterized bioactive peptides were derived from single domain peptide-encoding genes (Amano *et al.*, 2007; Ohyama *et al.*, 2008; Matsuzaki *et al.*, 2010; Meng *et al.*, 2012). However, a considerable number of regulatory peptide-coding genes, including CEPs, encode more than one peptide domain (Oelkers *et al.*, 2009; Delay *et al.*, 2013b; Imin *et al.*, 2013; Roberts *et al.*, 2013; Ogilvie *et al.*, 2014). Using the modified peptide isolation and enrichment protocol, mature 15-amino-acid bioactive peptides corresponding to both putative peptide domains of MtCEP1 (Fig. 2A, B) were isolated and identified from *MtCEP1ox* samples (Figs 2–3 and Supplementary Figs S1–3). The sequences of the eight domain 1 (D1) and one domain 2 (D2) species were determined (Fig. 2B) and the relative concentrations of each peptide were quantified using Quadrupole-Orbitrap MS. Five of the most abundant peptides identified in the *MtCEP1ox* sample were also identified in the vector control sample in low amounts (Fig. 2C). This is the first time that CEP peptides have been identified *in planta* without requiring constitutive or induced amplification of the peptide-encoding gene. The nano-LC-Chip-ESI-Q-TOF approach identified the five most abundant MtCEP1 species in the *MtCEP1ox* sample only (Supplementary Fig. S1).

The relative concentration of each peptide in the *MtCEP1ox* sample was determined by both mass spectrometers used and the ratio of each peptide species was consistent between biological replicates (Fig. 2D). The total peptide concentrations in the final concentrated extracts of *MtCEP1ox* and the vector control were 6.9 µM and 0.6 µM, respectively. Assuming minimal losses during isolation and concentration, the original peptide concentrations in the liquid culture root exudates were estimated to be 0.69nM and 60 pM, respectively (refer to Supplementary Method S2). The peptides isolated from the *MtCEP1ox* root culture eluted between 10 and 20min of the chromatographic run (Fig. 2E and Supplementary Table S1). The five peptides identified in the vector control sample were at trace levels with only two peptides producing discernible chromatographic peaks (Fig. 2F). For peptide identification and characterization, the Q Exactive Plus Orbitrap employed a full MS scan followed by top 10 data-dependant MS2 acquisition (Supplementary Method S1). Similarly the Q-TOF was run in full MS scan followed by auto-MS/MS with a preferred target list of the doubly charged precursor ions (Fig. 3, Supplementary Fig. S1 and Supplementary Method S2).

Among the nine peptide species identified, four proline-hydroxylated variants corresponded to the D1 peptide. Hydroxylation occurred at Pro11 (D1:HyP11), Pro4 and Pro11 (D1:HyP4,11), Pro7 and Pro11 (D1:HyP47,11) and, Pro4, Pro7 and Pro11 (D1:HyP4,7,11). From the MS/MS spectra, the three signature peaks for the D1 peptide variants (y5, y9, and y12) were used to determine the hydroxylation at the three proline residues as shown by the D1:HyP4,7,11 spectrum (Fig. 3A, Supplementary Fig. S3 and Supplementary Method S3). Another four D1 peptide variants were identified as having triarabinosylation at Pro11. These peptides were the arabinosylated counterparts of the four hydroxylated D1 peptides (Fig. 2B, Fig. 3, Supplementary Fig. S4 and Supplementary Method S3). The peptides were identified with triarabinosylation at Pro11 (D1:TaP11), hydroxylation at Pro4 and triarabinosylation at Pro11 (D1:HyP4,TaP11), hydroxylation at Pro7 and triarabinosylation at Pro11 (D1:HyP7,TaP11), and hydroxylation on both Pro4 and Pro7 with triarabinosylation at Pro11 (D1:HyP4,7,Tap11). Similarly, the three signature peaks were used to determine the respective triarabinosylation position on the proline residue (Fig. 3B and Supplementary Fig. S4). For the D2 peptide, only one species was identified with hydroxylation at Pro11 (Fig. 3C). The hydroxylated peptides constituted 93.5% of the total peptides isolated while the triarabinosylated peptides constituted only 6.5% of the total.

### MtCEP1 peptide species with specific hydroxylation patterns affect root development differentially

**Fig. 4.**
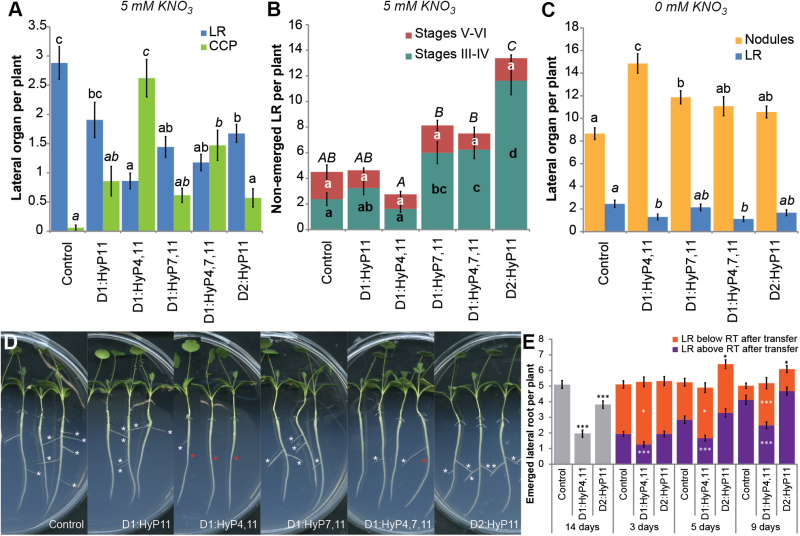
The effects of different synthetic MtCEP1 peptide species on lateral organ development on wild-type (A17) roots. (A) Emerged lateral root and CCP site formation was measured on roots grown on Fåhraeus medium containing 5mM KNO_3_ and different species of MtCEP1 peptides; (n ≥ 21). (B) A17 roots grown on different MtCEP1 peptides were counted after root clearing for non-emerged lateral roots at Stages III–IV and V–VI; (n ≥ 8). (C) Nodules and emerged lateral roots were observed on roots inoculated with *S. meliloti* WSM1022 and grown with the respective MtCEP1 peptides; (n ≥ 26). For (B-C), statistically significant differences were determined with ANOVA (F < 0.001), followed by a Tukey’s post-hoc analysis test at the 95% confidence level (α = 0.05) indicated by the lettering. In (B), the lowercase letters indicate the significance level of either stages III–IV or V–VI and the uppercase letters indicate the significance level for the total number of lateral root primordia. (D) Lateral root formation on representative plants grown on 5mM KNO_3_ in the presence of different peptides. White and red asterisks indicate emerged lateral roots and CCP sites, respectively. (E) Effects of temporal CEP peptide exposure on the lateral root formation. Lateral root emergence was quantified on 14-day-old plants exposed to either synthetic D1:HyP4,11 or D2:HyP11 for three, five or nine days before transferring the plants to a medium without peptide. Plants exposed to D1:HyP4,11 or D2:HyP11 or no peptide addition for the entire 14 days are included (no transfer, grey). Immediately after the transfer, the root tip (RT) position was marked. The lateral root numbers forming above RT after transfer or forming below RT after transfer (grown in the absence of CEP peptide) were scored. n ≥ 30; *, *P* ≤ 0.05; ***, *P* ≤ 0.001 (Student’s *t*-test). Error bars represent standard error.

To determine the biological effects of the different MtCEP1 peptide PTMs, the hydroxylated peptide isoforms were synthesized and assayed since these were the most abundant species found. It should be noted that synthetic Fmoc derivatives of triarabinosylated proline are not commercially available and this precludes the ability to make peptides with triarabinosylated prolines. Lateral root and CCP site formation were assessed at 5mM KNO_3_ (Fig. 4A, B, D); nodule formation was assessed at 0mM KNO_3_ (Fig. 4C). As expected, most peptide variants inhibited emerged lateral root number and induced periodic CCP sites (Fig. 4A, D). The D1:HyP4,11 showed the strongest inhibition of lateral root number and induced the highest number of CCP sites. The most abundant domain 1 peptide isolated from the root exudates, D1:HyP4,7,11, imparted less prominent phenotypes than D1:HyP4,11. A titration of D1:HyP4,7,11 showed significant inhibition of lateral root emergence at concentrations as low as 10^–9^ M (Supplementary Fig. S5).

A root clearing procedure was developed to determine the effects of the MtCEP1 peptide species on the early stages of lateral root formation defined by Herrbach *et al.* (2014) using differential interference contrast microscopy. Since *M. truncatula* roots are over 15 times the diameter of *Arabidopsis* roots, the early stages (I and II) of lateral root formation were impossible to observe and count due to the limited cellular resolution resulting from the thick root. However, stages III–IV and V–VI can be scored (these were pooled due to the limited ability to distinguish between the two paired stages). At 5mM KNO_3_, D1:HyP4,11 inhibition of emerged lateral root number was not linked to any significant changes at stages III–IV and V–VI (Fig. 4B). However, D1:HyP7,11, D1:HyP4,7,11 and especially the second domain D2:HyP11 peptides significantly increased lateral root formation at stages III–IV (3-fold for D1:HyP7,11 and D1:HyP4,7,11 and 6-fold for D2:HyP11; Fig. 4B; *P* < 0.001). This differential activity suggests that there are at least two distinct functional classes of MtCEP1 peptides.

As *MtCEP1* is up-regulated by nitrogen limitations (Imin *et al.*, 2013), the bioactivity of the MtCEP1 peptides was also tested for lateral root inhibition and enhanced nodule formation at 0mM nitrate. D1:HyP4,11 and D1:HyP7,11 significantly increased nodule number with D1:HyP4,11 forming the highest nodule number. Interestingly, under these conditions only D1:HyP4,11 and D1:HyP4,7,11 inhibited lateral root formation (Fig. 4C).

### The effects of continuous versus transient exposure of CEP peptides on root development

Since MtCEP1 D1:HyP4,11 induced the strongest inhibition of emerged lateral root number and D2:HyP11 induced the highest number of lateral root initiations, these peptides were studied further. We examined whether removal of peptide exposure rescued the inhibition of lateral root formation by D1:HyP4,11 or promoted the emergence of lateral root primordia induced by D2:HyP11. This was done by transferring the plants initially grown with the respective peptides for three, five or nine days respectively to growth medium without peptides and plants were grown for 14 days in total (Fig. 4E). The root tips at the time of transfer were marked to delineate the root region that had been exposed to the peptide from the region that grew subsequent to peptide removal. Control plants were grown with continuous exposure to the peptide or no peptide exposure for 14 days (Fig. 4E). Continuous exposure to D1:HyP4,11 and D2:HyP11 peptides (no transfer) reduced the number of emerged lateral roots significantly. For D1:HyP4,11, the root region grown on the peptide for three, five or nine days respectively formed a significantly lower number of emerged lateral roots. Upon peptide removal, the total emerged lateral root number was restored to the wild-type level due to an increase in emerged lateral root number from the new root regions that grew in the absence of peptide exposure (Fig. 4E). For D2:HyP11, there was no significant change in emerged lateral root number on the root regions exposed to the peptide for three days, thus the total number of emerged lateral roots was similar to the non-treated control. However, when exposed to D2:HyP11 for five or nine days, the total number of emerged lateral roots was higher compared to the non-treated control. Thus for D2:HyP11, the peptide removal promoted lateral root emergence only after more than five days’ exposure to the peptide.

### Interactions of MtCEP1 D1:HyP4,11 and D2:HyP11 peptides with auxin treatment and response

**Fig. 5.**
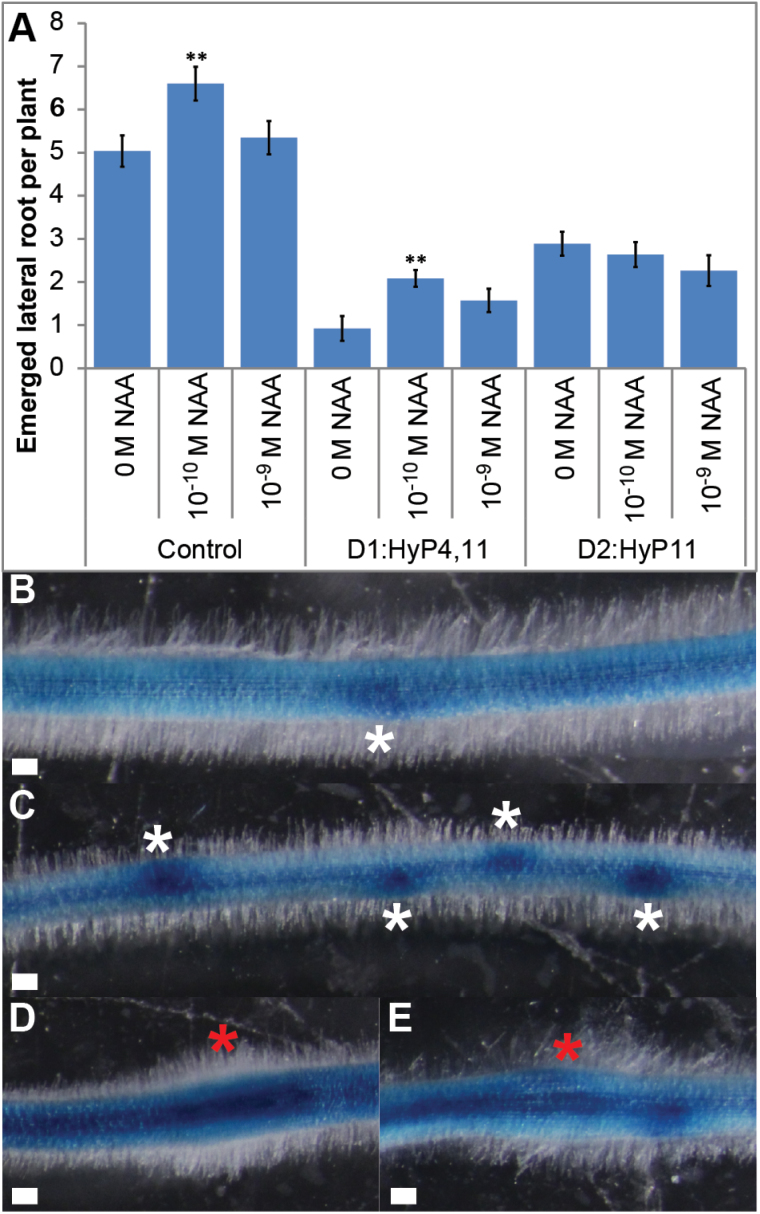
Interactions of MtCEP1 peptides with NAA treatment and CEP peptide effect on *GH3:GUS* expression in roots. (A) Lateral root formation on 14-day-old plants grown on medium with or without synthetic D1:HyP4,11 or D2:HyP11 in the presence of NAA at 10^–9^ M or 10^-10^ M or 0M as the control. (Student’s t-test; **, *P* ≤ 0.01; n ≥ 16). (B) Non-treated control roots showing the formation of young lateral root primordium expressing *GH3:GUS* (white asterisk). (C) The D2:HyP11 peptide which stimulated the formation of lateral root primordia showed strong staining (white asterisks). (D, E) CCP sites (red asterisks) induced by the D2:HyP11 (D) and D1:HyP4,11 (E) are shown. Scale bars, 100 µm.

NAA stimulates lateral root initiation by specifying lateral root founder cells (Casimiro *et al.*, 2001; De Smet *et al.*, 2007; Dubrovsky *et al.*, 2008) and promotes lateral root emergence or both initiation and emergence in *Arabidopsis*, rice and tobacco (Bhalerao *et al.*, 2002; Campanoni and Nick, 2005; Sreevidya *et al.*, 2010). Therefore, NAA was assessed for its ability to override the inhibitory effects on lateral root formation of these two peptides (Fig. 5A). Using a titration assay, we determined that the optimal NAA concentration for stimulating lateral root emergence in *Medicago* without inhibiting the primary root growth was 10^-10^ M (Supplementary Fig. S6). Therefore, the effects of adding NAA at 10^-10^ or 10^–9^ M were assessed. NAA at 10^-10^ M significantly increased the number of emerged lateral roots in control samples. NAA (10^-10^ M) in the presence of D1:HyP4,11 induced a small but significant increase in the number of emerged lateral roots compared to roots treated only with the peptide, however the overall level was over three-fold lower than 10^-10^ M NAA treatment alone. Further raising NAA to 10^–9^ M did not significant increase lateral root number. NAA addition at 10^-10^ M or 10^–9^ M did not increase the number of emerged lateral roots on plant grown with D2:HyP11.

To determine whether the D1:HyP4,11 and D2:HyP11 peptides affect root auxin distribution, we used the auxin-responsive *GH3:GUS* reporter construct that is commonly used to infer changes in auxin distribution and sensitivity in legumes (Larkin *et al.*, 1996; Mathesius *et al.*, 1998). In the control, lateral root primordia were prominently stained (Fig. 5B, white asterisks). For both peptide treatments, stronger staining was observed in the vascular tissues (Fig. 5C–E). Additionally, consistent with results in Fig. 4B, a high number of young lateral root primordium sites were induced by D2:HyP11 and these were also prominently stained (Fig. 5C). Again consistent with Fig. 5A, roots grown with D1:HyP4,11 showed strong inhibition of lateral root initiation and preferential formation of periodic CCP sites (Fig. 5D, red asterisks). The CCP sites showed strong localized *GH3:GUS* staining in the underlying vascular tissue and the surrounding inner cortical cells (Fig. 5D and Supplementary Fig. S6B) compared to the control (Fig. 5B and Supplementary Fig. S6A). The D2:HyP11 peptide induced a similar staining pattern at CCP sites (Fig. 5E and Supplementary Fig. S6B). Roots transformed with the vector control showed similar staining pattern as roots grown with no peptide (Supplementary Fig. S6C, D). *MtCEP1ox* roots showed increased staining in the vascular tissue and intense localized staining at CCP sites (Supplementary Fig. S6D).

## Discussion

Most biologically active secreted peptides acquire PTMs *in vivo*. These modifications can elevate activity up to 1000 times higher than their non-modified counterparts (Ohyama *et al.*, 2009; Matsuzaki *et al.*, 2010; Whitford *et al.*, 2012). Therefore, defining the full range of mature peptides with their PTMs produced *in vivo* is important to study their full biological activities. To address this, most protocols use cultures overexpressing peptide coding genes of interest to maximize production. In this study, we devised a more optimized extraction, enrichment and identification procedure to define several mature forms of secreted MtCEP1 peptides in root cultures overexpressing *MtCEP1* or containing the vector control. The modified protocol developed in the present study was robust and resulted in a 10 000-fold concentration of peptides in root culture exudates, reproducible detection of peptides by two types of mass spectrometers, and congruent results being obtained with independent biological preparations. Using this procedure we identified five hydroxylated CEP peptides and four novel triarabinosylated species as well as peptides corresponding to both domains of MtCEP1. We demonstrated that the identification of low abundant arabinosylated peptides requires an effective peptide isolation protocol and highly sensitive MS. This is the first study that identifies triarabinosylation of CEP peptides.

The identification of novel post-translationally modified MtCEP1 peptides corresponding to the two MtCEP1 domains suggests that maturation of CEP peptides is complex. Hydroxylated peptides comprised over 93% of MtCEP1 peptides compared to the arabinosylated peptides (~6%). The D1:HyP4,7,11 comprised 54% of the total isolated peptides found. Since these abundant hydroxylated peptides were also present in the vector control, this indicates that they are very likely to occur *in vivo.* As proline hydroxylation is a requirement prior to arabinosylation by hydroxyproline O-arabinosyltransferase (HPAT) (Ogawa-Ohnishi *et al.*, 2013), it is likely that the HPATs are the limiting factor for arabinosylating the peptides. This could be due to HPATs being infrequently co-located with the proline hydroxylase enzymes, or HPATs being expressed under different conditions to the hydroxylating enzymes. Only the Pro11 of the D1 peptides was triarabinosylated. The strong conservation of CEP *C*-terminal residues that occurs widely across species (Delay *et al.*, 2013b; Imin *et al.*, 2013; Ogilvie *et al.*, 2014) may be needed for the enzymatic modification of Pro11. Imin *et al.* (2013) demonstrated that CEPs were required to be 15-amino-acids and hydroxylated for biological activity.

The five peptides identified in the vector control samples were also the five most abundant peptides in *MtCEP1ox* samples but they were present in the latter at ~10 times lower concentration. Therefore, this CEP peptide composition is likely to reflect the endogenous CEP composition of *M. truncatula* roots. The 500-fold higher expression of *MtCEP1* relative to the native gene (Imin *et al.* 2013) leads to demonstrable root phenotypes and enables CEP activity to be bioassayed. However, the CEP peptides found in this study are not at 500 times the level of those of the empty vector control. One possible explanation is that the higher root mass of vector control samples may have compensated the lower *MtCEP1* expression compared to the *MtCEP1ox* samples. Alternatively, the higher root mass of the control roots might also have led to faster nitrogen depletion, which, in the roots containing the vector control, may have induced native *MtCEP1* expression sufficiently to be detected by MS. Nitrogen depletion is known to significantly elevate *MtCEP1* expression (Imin *et al.*, 2013).

The difference in the degree and position of the hydroxylation moieties on the D1 peptides resulted in different effects on root development. In Imin *et al.* (2013), roots overexpressing *MtCEP1* imparted strong inhibition of emerged lateral root number, enhancement of root nodule formation and induction of periodic CCP sites. These results appear to reflect the particularly strong effects that the D1:HyP4,11 peptide has for inhibiting lateral root emergence, enhancing nodulation and inducing CCP sites.

The peptide removal experiments were designed to determine if the exposure of CEP peptides imparted transient or long lasting effects on lateral root development. The removal of D1:HyP4,11 peptide exposure (Fig. 4E) did not rescue the inhibition of lateral root emergence in the root regions exposed to the peptide. However, a compensatory higher number of lateral roots was observed to emerge in the root regions that grew subsequently in the absence of the peptide. This demonstrates that these plants can restore the number of lateral roots to a wild-type level after the removal of D1:HyP4,11 peptide exposure. Similarly the strong inhibition of emerged lateral root number by D1:HyP4,11 was not restored to wild-type level even when NAA was added at optimal concentrations to promote lateral root emergence. Therefore D1:HyP4,11 seems to irreversibly inhibit lateral root emergence, possibly by perturbing the programming of lateral root formation.

In contrast to D1:HyP4,11, the domain 2 peptide, D2:HyP11 (as well as D1:Hyp7,11 and D1:Hyp4,7,11), significantly increased the number of non-emerged lateral root primordia at stages III–IV. This was corroborated by the increased number of non-emerged lateral roots observed by examining *GH3:GUS* expression in roots treated with D2:HyP11. Unlike the D1:Hyp4,11 peptide, the non-emerged lateral roots failed to grow further unless peptide exposure was removed, and this consequently led to increased lateral root emergence compared to control roots. NAA addition to wild-type plants at 10^-10^ M also induces a significant increase in the number of emerged lateral roots, as observed in other plants (Bhalerao *et al.*, 2002; Campanoni and Nick, 2005; Sreevidya *et al.*, 2010). However, NAA addition to D2:HyP11-treated roots did not change the emerged lateral root number. Therefore, the high number of lateral root primordia at stages III–IV induced by D2:HyP11 most likely requires an auxin independent pathway(s) to promote their emergence in *M. truncatula*.

CEP peptides may influence auxin level, sensitivity or flow since they alter the root expression pattern of the auxin-responsive *GH3:GUS* reporter. The strong GUS staining observed at CCP sites especially in the presence of D1:HyP4,11 correlates with the increase in cell division previously observed at these sites (Imin *et al.*, 2013). The stronger staining of *GH3:GUS* observed in the vascular tissues of peptide-grown roots may reflect a supraoptimal auxin response which could affect lateral root development (De Smet *et al.*, 2007). However, although auxin maxima correlates with lateral root formation as reflected by the strong *GH3:GUS* staining at CCP sites, this was not sufficient to encourage lateral root formation in the presence of D1:HyP4,11.

The distinctive biological activities of the different MtCEP1 peptide species could be due to the differential perception and recognition of specific peptides. NMR analysis of MtCEP1 (D1:HyP4,11) and a root-knot nematode CEP revealed that hydroxylation of Pro4 and Pro11 resulted in lower structural constraints on the peptide backbone (Bobay *et al.*, 2013). This may reflect the different biological effects imparted by MtCEP1 peptides with different proline hydroxylation patterns. Other modifications such as arabinosylation and sulfation strongly alter biological activities of CLE and RGF peptides, respectively (Shinohara and Matsubayashi, 2013). The structural differences resulting from these PTMs could provide binding specificity of the peptides to their respective receptor(s).

In conclusion, our modified extraction and isolation protocol enabled sufficient enrichment of endogenous peptides which led to the detection, identification and quantification of nine MtCEP1 peptide species including novel arabinosylated CEP peptide species using a sensitive MS analysis. This suggests that MtCEP1-mediated regulation of root development results from the processing and PTM of various peptide species and/or multi-domain peptides from a single gene. The differential activity of these CEP peptides for regulating root development might be attributable to the differences observed in the PTMs. Hence, the complexity of peptide-mediated developmental processes is influenced by the primary sequence of the peptide and its processing and post-translational modification. Future work characterizing more endogenous secreted peptides is essential to understand how these potent regulatory molecules are processed and modified, and how these modifications influence their biological activities. Our improved procedure for peptide isolation should provide a robust and efficient method to identify and characterize other secreted peptides. Our work also shows that the inhibition of lateral roots and the formation of periodic CCP sites occur in root cultures overexpressing *MtCEP1* in the absence of intact shoots and light. Therefore, CEP peptides may also mediate local responses in the root in addition to the systemic responses recently identified by Tabata *et al.* (2014).

## Supplementary Material

Supplementary data can be found at *JXB* online.

Supplementary Fig. S1. Extracted ion chromatographic (EIC) separation and MS/MS spectra of the five peptides identified in *MtCEP1ox* sample with nano-LC-ESI ChipCube ion source Q-TOF.

Supplementary Fig. S2. MS/MS spectra of MtCEP1 hydroxylated peptides using the Q Exactive Plus Orbitrap MS.

Supplementary Fig. S3. MS/MS spectra of MtCEP1 triarabinosylated peptides using the Q Exactive Plus Orbitrap MS.

Supplementary Fig. S4. Titration of biological activity of the MtCEP1 D1:HyP4,7,11 peptide for inhibition of lateral root and induction of CCP site formation.

Supplementary Fig. S5. Titration of NAA on *M. truncatula* plants to determine the optimal concentration for stimulating lateral root emergence without inhibiting primary root growth.

Supplementary Fig. S6. Staining of *GH3:GUS* on cross-sections of peptide-treated roots and transformed hairy roots.

Supplementary Method S1. Q Exactive Orbitrap Targeted-SIM-ddMS2.

Supplementary Method S2. Identification and quantification by nano-LC-Chip-ESI-MS/MS.

Supplementary Method S3. Determining the positions of post-translational modifications based on peptide fragmentation from MS/MS spectra acquired using the Q Exactive Plus Orbitrap MS.

Supplementary Table S1. List of identified peptides from *MtCEP1ox* sample with the retention time (tR) of the doubly charged ion [M+2H]^2+^.

Supplementary Data
