# Supplementary material for: Novel MtCEP1 peptides produced in vivo differentially regulate root development in Medicago truncatula
Source: J Exp Bot. 2015 Feb 22;66(17):5289–300. doi: 10.1093/jxb/erv008 (PMC4526912; doi:10.1093/jxb/erv008)
Supplement: Supplementary Data [file supp_66_17_5289__index.html]

Novel MtCEP1 peptides produced in vivo differentially regulate root development in Medicago truncatula — Supplementary Data 

# Novel MtCEP1 peptides produced *in vivo* differentially regulate root development in *Medicago truncatula*

## Supplementary Data

Data files

**Files in this Data Supplement:**

- Supplementary Data - Supplementary Data
- Supplementary Data - Supplementary Data
