# Supplementary material for: Novel MtCEP1 peptides produced in vivo differentially regulate root development in Medicago truncatula
Source: J Exp Bot. 2015 Feb 22;66(17):5289–300. doi: 10.1093/jxb/erv008 (PMC4526912; doi:10.1093/jxb/erv008)
Supplement: Supplementary Data [file supp_erv008_JXBRevised_Supplementary_information_150115.pdf]

Supplementary Information

Supplementary Method 1. Q Exactive Orbitrap Targeted-SIM-ddMS2.

Supplementary Method 2. Identification and quantification by nano-LC-Chip-ESI-MS/MS.

Supplementary Method 3. Determining the positions of post-translational modifications based on peptide fragmentation from MS/MS spectra acquired using the Q Exactive Orbitrap MS.

Supplementary Table 1. List of identified peptides from *MtCEP1ox* sample with the retention time (tR) of the doubly charged ion $(M+2H]^{2+}$.

Supplementary Method

1 Q Exactive Orbitrap Targeted-SIM-ddMS2

A scheduled targeted SIM-ddMS2 method was investigated on a refined (shortened) inclusion list of 7 peptide precursor ions for HRAM (high resolution accurate mass) relative quantification of selected peptides. The method implemented a 4 Dalton mass window for SIM scans at 70 K resolution and 35 K resolution for high energy collision dissociation (HCD) ddMS2 scans with an AGC (Automatic Gain Control) target setting of 2e5 counts and 1e5 counts for SIM and ddMS2 scans respectively. Maximum fill times of 240ms (SIM) and 110 ms (ddMS2) were used while an intensity threshold of 1.4e4 counts was set for triggering the HCD ddMS2 scan events with a dynamic exclusion of 10 seconds. The normalized collision energies were as specified in the table:

Precursor Ion [m/z]	Molecular Formula / Sequence	Charge State [z]	Ion Polarity	Peak Start [min]	Peak End [min]	NCE (eV)
749.35651	Synthetic peptide as standard ($C_{64}H_{96}N_{20}O_{20}$)	2	Positive	13.1	16.14	26
741.85223	$C_{64}H_{95}N_{19}O_{22}$	2	Positive	16.17	21	26
749.84969	$C_{64}H_{95}N_{19}O_{23}$	2	Positive	16.17	21	26
757.84714	$C_{64}H_{95}N_{19}O_{24}$	2	Positive	13.1	16.14	26
804.38169	$C_{69}H_{102}N_{22}O_{23}$	2	Positive	9	13.08	27
947.91307	$C_{79}H_{119}N_{19}O_{35}$	2	Positive	16.17	21	28
955.91053	$C_{79}H_{119}N_{19}O_{36}$	2	Positive	16.17	21	28

The extracted ion chromatogram (+/- 5ppm, XIC) for each precursor m/z included in the target list was used for calculating the relative concentration of each peptide measured in the exudate extracts against the calibration curve of the synthetic peptide standard that ranged from 20 amol to 200 fmol. The tSIM-ddMS2 method parameters used for acquiring the standard curve data were as previously described with the following modifications. Resolution was set to 140 K with a maximum fill time of 500 ms. A mass window of 50 Da was used for the SIM scan and an intensity threshold of 1.8e4 counts was set for triggering the HCD ddMS2 scan events with a dynamic exclusion of 10 seconds. Peptide ID was confirmed via MS/MS fragment spectral matching against an annotated *Medicago truncatula* protein database to which was added the 4 MtCEP1 peptides performed with Proteome Discoverer 1.4.

2 Identification and quantification by nano-LC-Chip-ESI-MS/MS

MtCEP1ox, vector control and synthetic peptide samples were analysed using an Agilent 6530 Accurate-Mass Q-TOF LC/MS system (Agilent Technologies, Inc., Santa Clara, CA, USA) with a ChipCube ion source MS interface (Agilent Technologies, Inc., Palo Alto, CA)

utilising a liquid chromatographic chip (ProtID-Chip-150(II), comprising an enrichment column: 4 mm 40 nL, and an analytical separation column: 150 mm x 75 μm; packed with 5 μm Zorbax 300SB-C18 particles). The LC separation used two pumps: a binary capillary pump operated at a flow rate of 4 μL/min, used for loading the samples, and a nanoflow gradient pump using a linear gradient from 8 (hold 2 min) to 38% mobile phase B in 47 min at a flow rate of 300 nL/min. The column was then washed with 90% mobile phase B for 5 min. Mobile phase A was 0.1% formic acid in water and mobile phase B was 90% acetonitrile/water with 0.1% formic acid. The same mobile phases were used for both the capillary and nanoflow pumps. The sample was solubilized in 20 μL of 10% acetonitrile/water with 0.1% formic acid and a 7 μL sample aliquot was injected.

The nanospray from the HPLC-Chip was subjected to positive ion polarity ESI using the following settings: gas flow rate 4 L/min, gas temperature 300°C, capillary voltage 1900 V, fragmentor 175 V, skimmer 65 V and octopole RF peak 750 V. The instrument was run in extended dynamic range mode at 2 GHz with data dependent acquisition switching between MS (m/z 110 – 3200 at 2 spectra/s) and auto MS/MS (m/z 50 – 3200 at 3 spectra/s) with a preferred list targeting specified doubly-charged peptides. Collision induced dissociation (N2 collision gas supplied at 18 psi, medium 4 amu isolation window) was done on fragment spectra of the 8 most intense precursor ions with charge states 2, 3 and ≥ 3 with a 15 s dynamic exclusion time, within a cycle time of 3.249 s. The collision energy was automatically set by the Agilent MassHunter Acquisition software (using the formula: $CE = \frac{Slope \times (m/z)}{100 + Offset}$, where slope 3, offset 2). The m/z values of all ions present in the mass spectra were corrected against two reference ions (purine, [MH]+ m/z 112.9856 and 1H, 1H, 3H tetra(fluoropropoxy)phosphazine, [MH]+ m/z 922.0097). Data was acquired and analysed with Agilent Technologies MassHunter software (version B.5.0).

To quantify each peptide in the root culture liquid extract, a calibration curve using synthetic peptide dilutions was constructed (10 to 100 µM). Aliquots of the sample were injected into the nano-LC-Chip-ESI-QTOF and the peak areas acquired were used to determine the relative concentration of each peptide species in the original root exudate liquid culture.

3 Determining the positions of post-translational modifications based on peptide fragmentation from MS/MS spectra acquired using the Q Exactive Orbitrap MS

For the hydroxylated peptides, the doubly charged ions $[M+2H]^{2+}$ were targeted for their MS/MS fragmentation pattern. The four hydroxylated D1 peptide isoforms successfully identified were: (1) D1:HyP4,7,11 hydroxylated at all three prolines (757.847 m/z), (2) D1:HyP4,11 hydroxylated on two prolines, Pro4 and Pro11 (749.850 m/z), (3) D1:HyP7,11 with hydroxylation on Pro7 and Pro11 (749.850 m/z) and (4) D1:HyP11 hydroxylated only at Pro11 (741.852 m/z). For D2 peptides, one isoform was found to be hydroxylated at Pro11. As with the arabinosylation site, the three proline signature peaks were observed by a characteristic 16 mass unit difference depending on the degree of hydroxylation on the proline residue. For example, the hydroxylation on D1:HyP4,7,11 (Figure 3B) at all three prolines will shift the 466.241 m/z to 482.235 m/z, the 837.384 m/z to 853.378 m/z and 1152.528 m/z to 1168.521 m/z. The same mass difference was used to determine the position of the two hydroxyprolines for the two isoforms with the same mass; D1:HyP4,11 and D1:HyP7.11. Similarly, the 16 mass unit difference enabled the monohydroxylation to be located on Pro11 for D1:HyP11 (Supplemental Figure 2). The MS/MS fragmentation patterns of the monohydroxylated D2 peptide also showed hydroxylation at Pro11. The five hydroxylated peptide species identified with the Q-TOF also showed the same three signature

ion peaks which confirmed the post-translational modifications on the five peptides (Supplementary Figure 1).

For the arabinosylated peptides, the identified peptides were: (1) D1:HyP4,7,TaP11; D1 peptide with hydroxylation on Pro4 and Pro7 and triarabinosylation at the Pro11 with a peak eluting at 16.11 min (955.911 m/z), (2) D1:HyP4,TaP11; D1 peptide with hydroxylation on Pro4 and triarabinosylation at Pro11 with a peak at 17.90 min (947.913 m/z), (3) D1:HyP7,TaP11; D1 peptide with hydroxylation on Pro7 and triarabinosylation at Pro11 with a peak at 18.79 min (947.913 m/z) and (4) D1:TaP11; D1 peptide with only arabinosylation at Pro11 eluting at 20.98 min (939.916 m/z). Again, the MS/MS fragmentation of MtCEP1 peptides produced three strong signature peaks. These peaks corresponded to the y ion fragments from the peptide fragmentation at the three proline residues, y5, y9, and y12 respectively (Figure 3). For arabinosylation, the 132 m/z successive peaks following the three signature peaks corresponded to an arabinose molecule on a proline residue. For all four arabinosylated peptides, the successive peaks with 132 m/z difference started at the smallest fragment of the three, the y5 ion. For the D1:HyP4,7, TaP11 peptide with 955.911 m/z (Figure 3B), the MS/MS showed the y5 ion of 482.235 m/z was followed by the monoarabinose y5 ion of 614.277 m/z and successively by the diarabinose y5 ion of 746.317 m/z. Similar peak patterns were observed for y9 ion and y12 ion with the monoarabinose peaks of 985.420 m/z and 1300.563 m/z and the diarabinose peaks of 1117.459 m/z and 1432.612 m/z, respectively. As the larger y9 and y12 fragments consisted of the five residues of y5 ion (-PGVGH), the successive 132 m/z arabinose peaks had to originate from the Pro11 on the y5 fragment. Therefore, the triarabinose modification on D1 peptide occurred at the Pro11. The same fragmentation pattern with mono-arabinose peaks were observed for D1:HyP4,TaP11, D1:HyP7,Tap11 and D1:TaP11 (Supplementary Figure 3).

Supplementary Table 1: List of identified peptides from *MtCEP1ox* sample with the retention time (t_R) of the doubly charged ion $(M+2H]^{2+}$. The peptide sequence of each peptide is displayed in Figure 2.

Peptide	Molecular Formula	Molecular Weight (Da)	Precursor ion $[M+2H]^{2+}$	t_R (min)
D1:HyP11	$C_{64} H_{95} N_{19} O_{22}$	1481.6899	741.8522	20.42
D1:HyP4,11	$C_{64} H_{95} N_{19} O_{23}$	1497.6848	749.8497	17.32
D1:HyP7,11	$C_{64} H_{95} N_{19} O_{23}$	1497.6848	749.8497	18.23
D1:HyP4,7,11	$C_{64} H_{95} N_{19} O_{24}$	1513.6797	757.8471	15.31
D1:TaP11	$C_{79} H_{119} N_{19} O_{34}$	1877.8167	939.9158	20.98
D1:HyP4, TaP11	$C_{79} H_{119} N_{19} O_{35}$	1893.8116	947.9131	17.90
D1:HyP7, TaP11	$C_{79} H_{119} N_{19} O_{35}$	1893.8116	947.9131	18.79
D1:HyP4,7, TaP11	$C_{79} H_{119} N_{19} O_{36}$	1909.8065	955.9105	16.11
D2:HyP11	$C_{69} H_{102} N_{22} O_{23}$	1606.7488	804.3817	10.60
