# Supplementary material for: Novel MtCEP1 peptides produced in vivo differentially regulate root development in Medicago truncatula
Source: J Exp Bot. 2015 Feb 22;66(17):5289–300. doi: 10.1093/jxb/erv008 (PMC4526912; doi:10.1093/jxb/erv008)
Supplement: Supplementary Data [file supp_erv008_jexbot137778_file001.pdf]

Supplementary Figures

Supplementary Figure 1. Extracted ion chromatographic (EIC) separation and MS/MS spectra of the five peptides identified in *MtCEP1ox* sample with nano-LC-ESI ChipCube ion source Q-TOF.

Supplementary Figure 2. MS/MS spectra of MtCEP1 hydroxylated peptides using the Q Exactive Orbitrap MS.

Supplementary Figure 3. MS/MS spectra of MtCEP1 triarabinosylated peptides using the Q Exactive Orbitrap MS.

Supplementary Figure 4. Titration of biological activity of the MtCEP1 D1:HyP4,7,11 peptide for inhibition of lateral root and induction of CCP site formation.

Supplementary Figure 5. Titration of NAA on *M. truncatula* plants to determine the optimal concentration for stimulating lateral root emergence without inhibiting primary root growth.

Supplementary Figure 6. Staining of *GH3:GUS* on cross-sections of peptide-treated roots and transformed hairy roots.

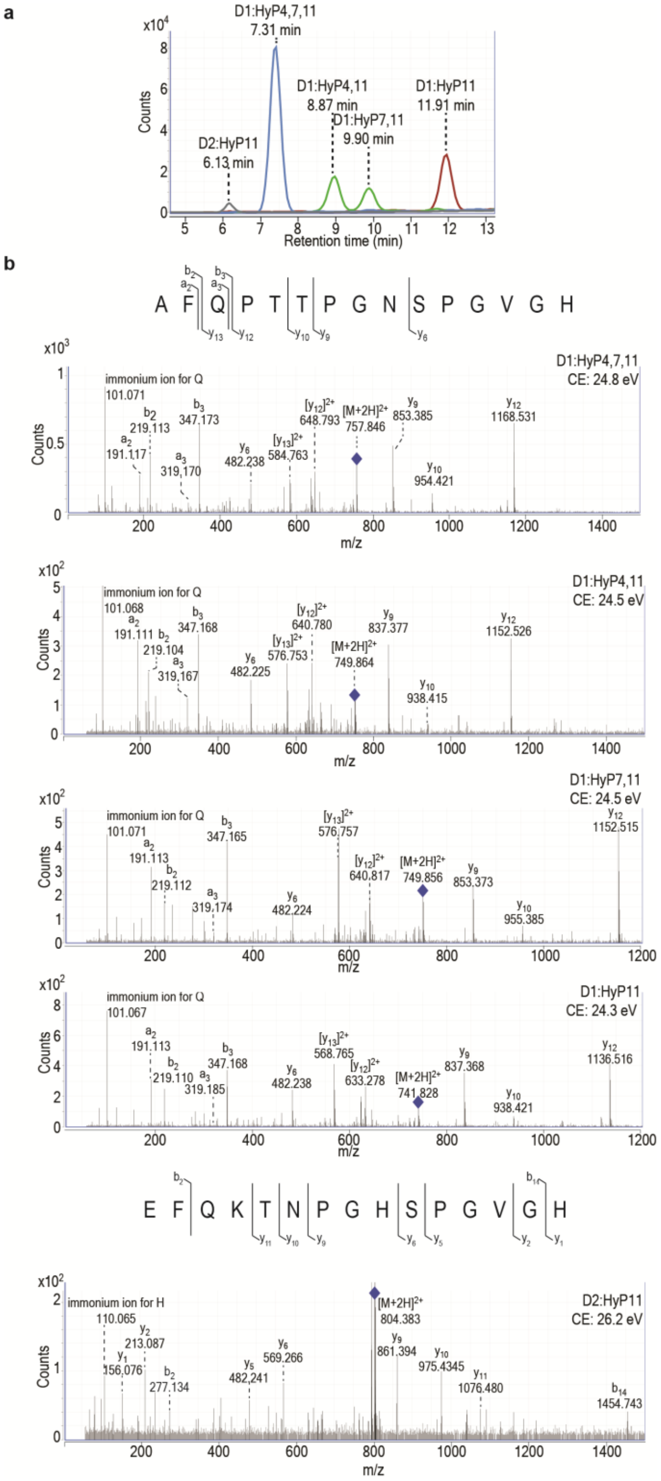

Supplementary Figure 1. Extracted ion chromatographic (EIC) separation and MS/MS spectra of the five peptides identified in *MtCEP1ox* sample with nano-LC-ESI ChipCube ion source Q-TOF. (a) The EIC chromatogram shows the respective retention times of the five peptides identified in the *MtCEP1ox* sample (b) The peptide sequence and hydroxylation positions were verified using full MS scan followed by auto-MS/MS. The doubly charged ions (indicated by the blue dots) were specifically targeted as precursor ions to produce the characteristic peptide fragmentation patterns for each peptide species (CE= collision energy).

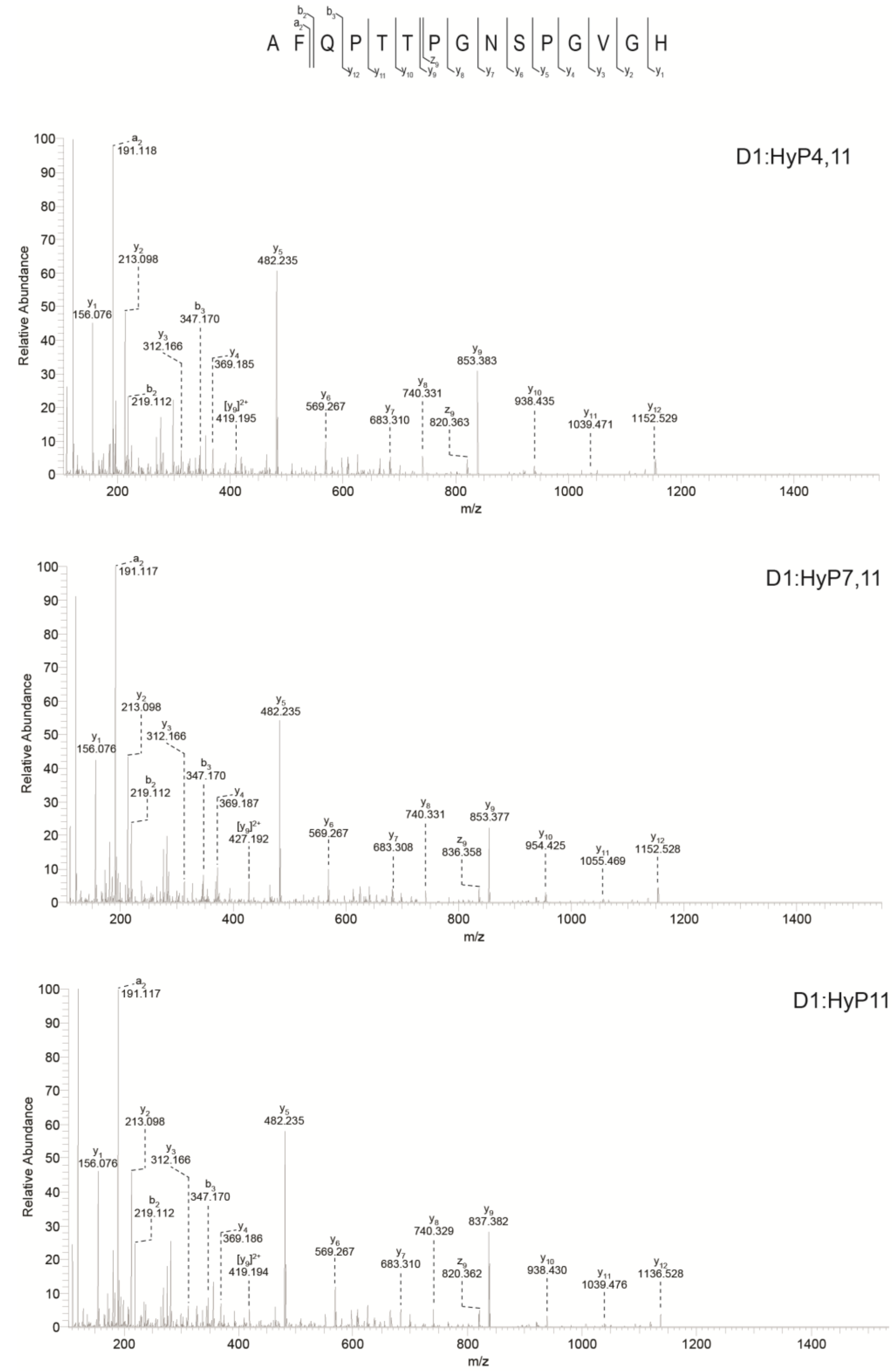

Supplementary Figure 2. MS/MS spectra for domain 1 of MtCEP1 hydroxylated peptides using the Q Exactive Orbitrap MS. From the three signature peaks of y5, y9, and y12, the hydroxylation position of the peptides were determined. The three peptides were hydroxylated at Pro4 and Pro11 (D1:HyP4,11), Pro7 and Pro11 (D1:HyP7,11) and Pro11 (D1:HyP11).

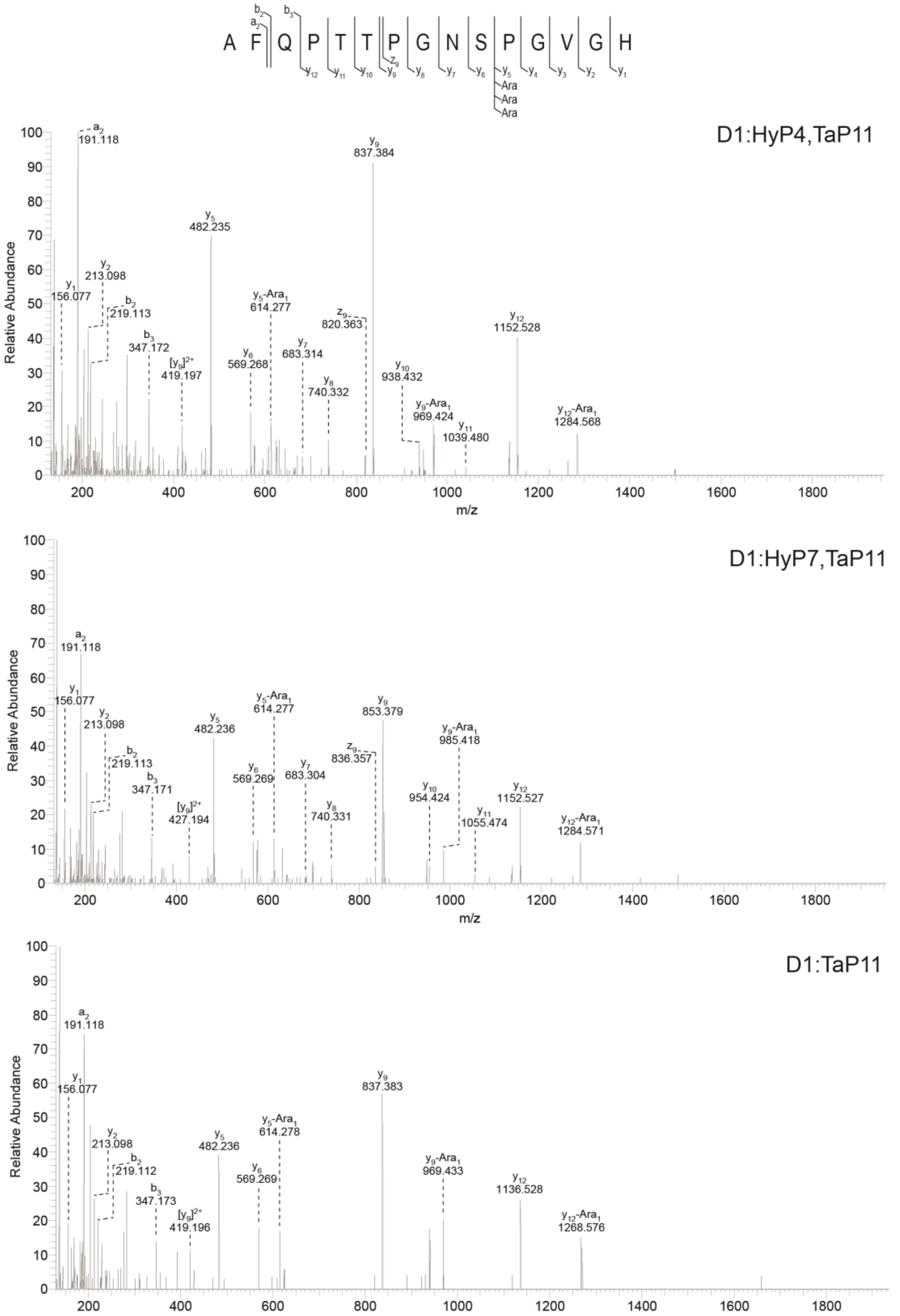

Supplementary Figure 3. MS/MS spectra for domain 1 of MtCEP1 triarabinosylated peptides using the Q Exactive Orbitrap MS. From the signature peaks of y5, y9 and y12, the triarabinose was determined at Pro11 in all three species. The different species identified differed by the positions of additional hydroxylation on Pro4 (D1:HyP4, TaP11) or Pro7 (D1:HyP7,TaP11). A triarabinosylated peptide without additional hydroxylation was also identified (D1:TaP11).

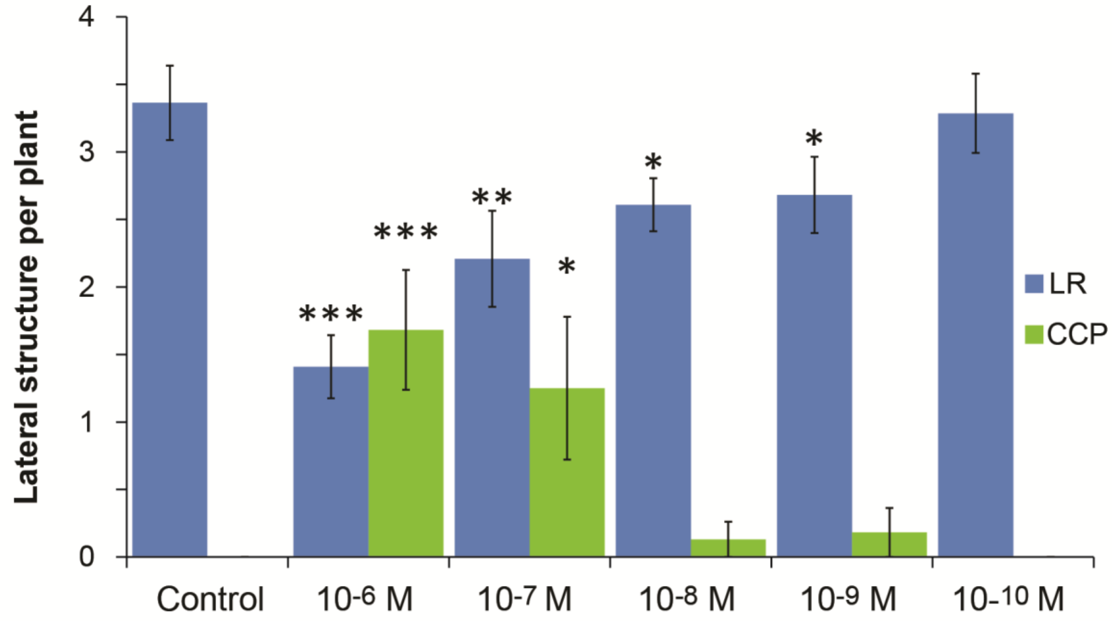

Supplementary Figure 4. Titration of biological activity of the MtCEP1 D1:HyP4,7,11 peptide for inhibition of lateral root and induction of CCP site formation. The lateral root inhibition is significantly inhibited between 10^{-6} M and 10^{-9} M whereas the induction of CCP formation is significant between 10^{-6} M and 10^{-7} M. Asterisks indicate significant differences (Student's t-test; *=P < 0.05; **=P <0.01; ***=P < 0.001) with N ≥ 22.

a

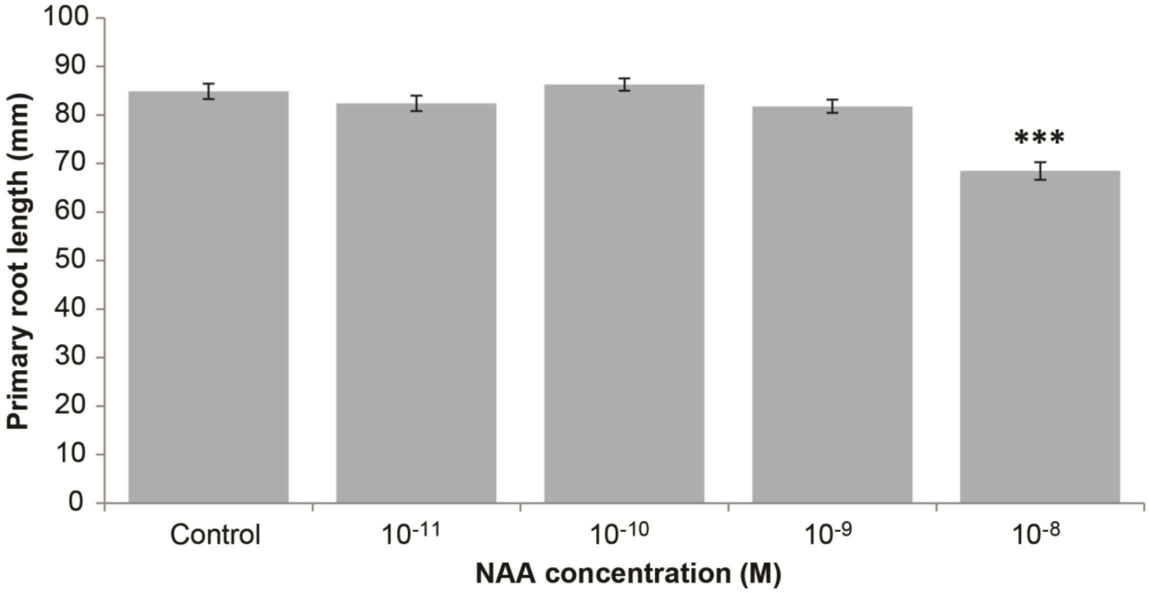

b

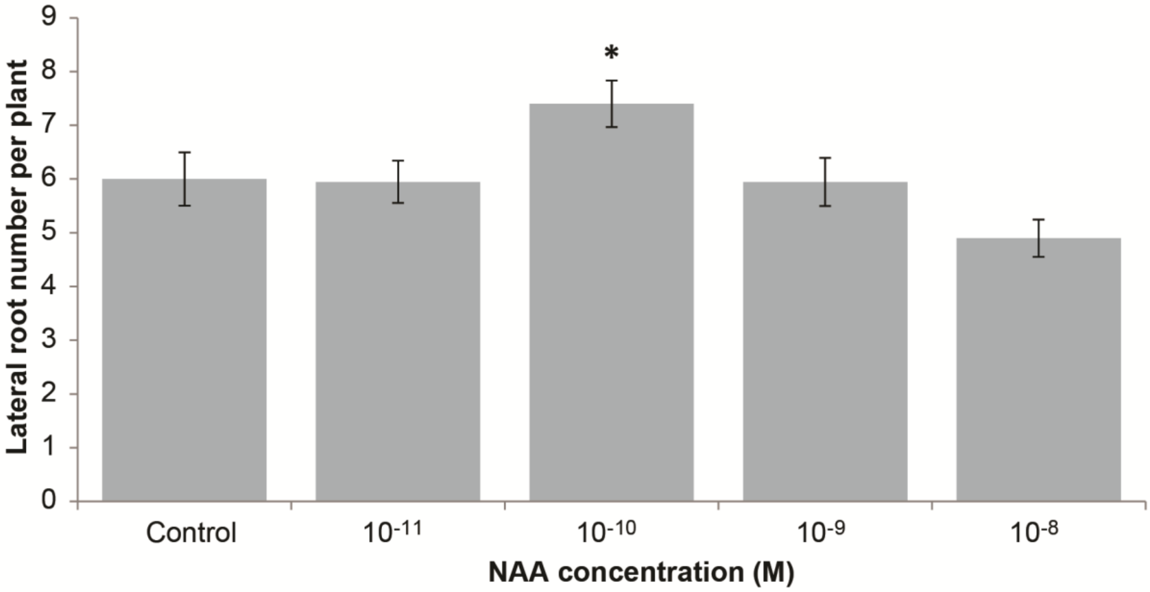

Supplementary Figure 5. Titration of NAA on *M. truncatula* plants to determine the optimal concentration for stimulating lateral root emergence without inhibiting primary root growth. (a) The NAA was titrated from 10^{-8} M to 10^{-11} M. At 10^{-8} M, the primary root was significantly inhibited. (b) The optimal concentration for stimulating lateral root emergence significantly was 10^{-10} M. Asterisks indicate significant differences (Student's t-test; *=P < 0.05; **=P <0.01; ***=P < 0.001) with N ≥ 18.

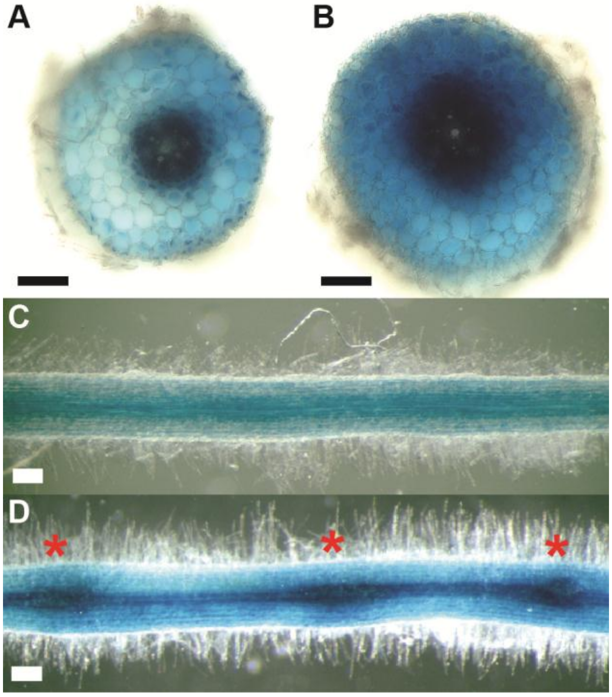

Supplementary Figure 6. Staining of *GH3:GUS* on cross-sections of peptide-treated roots and transformed hairy roots. (A,B) Vibratome sections of a non-treated (A) and D2:HyP11-treated (B) *GH3:GUS* root showing peptide-enhanced vascular staining. (Scale bar = 50 µm) (C,D) *GH3:GUS* expression in vector control(C) and *MtCEP1ox* roots (D). A strong *GH3:GUS* staining is detected at the CCP sites (red asterisks). (Scale bar = 100 µm)
